# Can Vıtamın D Reduce the Need for SSRI by Modulatıng Serotonın Synthesıs?: A Revıew of Recent Lıterature

**DOI:** 10.1007/s13668-025-00630-7

**Published:** 2025-03-01

**Authors:** Zekiye Zeynep Bostan, Melike Şare Bulut, Makbule Gezmen Karadağ

**Affiliations:** 1 Department of Nutrition and Dietetics, Faculty of Health Sciences, İstanbul Gedik University, Istanbul, Türkiye; 2 Department of Nutrition and Dietetics, Faculty of Health Sciences, Biruni University, Istanbul, Türkiye; 3 Department of Nutrition and Dietetics, Faculty of Health Sciences, Gazi University, Ankara, Türkiye

**Keywords:** Major depressive disorder, Neuropsychiatry, Serotonin, SSRIs, Vitamin D

## Abstract

**Purpose of Review:**

This study aims to analyze the effects of vitamin D on serotonin synthesis, release, and function in the brain, and to explore its relationship with various psychiatric disorders.

**Recent Findings:**

The hormone calcitriol plays a vital role in regulating a multitude of biological processes within the human body. Its deficiency can lead to significant adverse effects on overall health and well-being, including physical and psychological consequences. Evidence indicates that vitamin D may exert influences through receptors in the brain, modulating serotonin production and influencing emotional and cognitive processes. Recent studies propose that elevated serum vitamin D may enhance mood and alleviate depressive states. The impact of vitamin D on cognitive function and behavior remains an area of ongoing investigation. This literature review analyses the available evidence on how vitamin D intake impacts the severity of illness and medication requirements in diverse psychiatric disorders. A review of the literature suggests that there may be a correlation between vitamin D and serotonin, which could potentially contribute to more favorable outcomes in the context of illness. Vitamin D may increase the amount of serotonin in the synaptic gap, which is the intended use of selective serotonin reuptake inhibitors (SSRIs), with its effect on the increase in serotonin release.

**Summary:**

According to reports, vitamin D supplementation in conjunction with SSRI group medication provides an additive effect for the management of psychiatric disorders.

## Introduction

There is a large body of literature indicating that specific foods and dietary patterns can exert a pronounced impact on mental health outcomes. The mood of an individual has been demonstrated to influence their nutritional status, and food preferences, in conjunction with vitamin-mineral levels in the body, have also been shown to impact the mental state [1]. A substantial body of previous research has indicated a correlation between low levels of vitamin D and an increased likelihood of experiencing symptoms associated with depression and anxiety [2, 3]^.^

Vitamin D, which performs endocrine functions, exists in two forms, cholecalciferol (D3) and ergocalciferol (D2) [4, 5]. Calcitriol (1α,25-dihydroxyvitamin D3), a product of a cascade of biochemical reactions in diverse tissues, represents the biologically active form of vitamin D. Calcidiol (25(OH)D) is the primary circulating metabolite of vitamin D, with serum levels serving as the most dependable marker of vitamin D status [6].

For an extended period, it was assumed that the active form of vitamin D (1,25(OH)2D) could be produced exclusively in the kidneys through the conversion of 25(OH)D. Nonetheless, recent research has revealed that other tissues, including different regions of the brain (prefrontal cortex, hippocampus and hypothalamus), also exhibit the enzyme 1-alpha-hydroxylase, which converts 25(OH)D to 1,25(OH)2D, thereby indicating a crucial role of vitamin D in regulating cognitive and emotional processes [7].

The neurotransmitter serotonin has been shown to perform a crucial function in the regulation of a plethora of cerebral functions and behaviors, comprising executive process, selective attention, and social conduct. It has been implicated in attention deficit hyperactivity disorder, bipolar disorder, schizophrenia, impulsive behaviour, anxiety and depression. The primary treatment option for this condition is drug therapy using selective serotonin reuptake inhibitors (SSRIs). However, the extant evidence indicates that vitamin D exerts its effects on tissue-specific serotonin synthesis through a series of biological mechanisms**.**The production of serotonin takes place within the brain, with this neurotransmitter being formed from the amino acid tryptophan. The process is initiated by the enzyme tryptophan hydroxylase 2, which is activated at the transcriptional level by the hormone vitamin D. Several studies imply that vitamin D supplements could enhance clinical depressive status [8]. Nonetheless, the mechanism behind vitamin D supplementation's beneficial effects on cognitive function and behavior is still unclear [9–11].

The objective of this review is to present an analysis of the impact of vitamin D on the synthesis, release, and function of serotonin in the brain, as well as to examine the interrelationship between vitamin D, serotonin, and a range of psychiatric disorders. We will also analyze the mechanisms behind these effects.

## Vitamin D

Vitamin D, which was identified in the 20th Century, serves the dual function of acting as a precursor to hormones in addition to its classification as a fat-soluble vitamin [4, 5].

The derivation of D2 occurs through yeast, mushrooms, and similarly sourced items, whereas the synthesis of D3 encompasses a complex sequence of processes within the body. The process of vitamin D3 synthesis is initiated in the dermis by ultraviolet light, which induces the conversion of the precursor molecule, 7-dehydrocholesterol, into the prohormone cholecalciferol (1,24,25(OH)D3). Subsequently, vitamin D3 undergoes hydroxylation within the liver, resulting in calcidiole (25(OH)D3). Although 25(OH)D3 is biologically inactive, the enzyme 1α-hydroxylase converts it to calcitriol (1,25(OH)2D3), which is biologically active form. Initially, the enzyme was thought to be present only in the tubules of the kidney. However, studies have revealed that it is also expressed by various extrarenal tissues [12–14]^.^ While some vitamin D is obtained from nutrients such as liver, egg yolk, oily fish, etc. (10%), the bulk of the requirement (90%) is met via the conversion above pathway. Vitamin D metabolism is regulated by parathormone (PTH) as well as serum levels of calcium and phosphorus [12].

While the essential role of vitamin D in controlling bone metabolism and the regulation of calcium homeostasis is well-established, the discovery of the vitamin D receptor (VDR) in a multitude of organs and tissues indicates that this vitamin may have a more expansive function, potentially including the modulation of cell differentiation and proliferation [15]. Moreover, vitamin D has been associated with a number of physiological and pathological processes associated with a variety of diseases, including cancer, cardiac conditions, and metabolic syndrome [4]. Adequate vitamin D levels offer a range of benefits, including immune modulation and overall nervous system health, as well as ensuring efficient calcium metabolism [16]. Vitamin D receptors, which have the ability to regulate a multitude of genes and consequently modulate several cellular pathways, are expressed in nearly all human cells. They significantly impact the majority of vitamin D-related effects and are recognized for modulating approximately 3% of the human genome [4, 17].

Vitamin D plays a significant part in synthesizing, releasing and regulating neurotransmitters. Vitamin D insufficiency has been recorded among older adults, being linked to reduced grey matter volume in the hippocampus and calcarine sulcus, as well as a thinner cingulate cortex [18].

Individuals' serum vitamin D levels depend strongly on environmental factors related to their geographical region and lifestyle [4, 12]. Nevertheless, heritable factors also influence serum vitamin D levels, and there is evidence to suggest that they may contribute to the pathogenesis of several chronic diseases, encompassing osteoporosis, cancer, and diseases of the immune system. The most commonly studied genes in this context are DHCR7, CYP2R1, CYP27B1, GC, VDR, CYP24A1 and RXR [4].

Vitamin D insufficiency threatens almost 50% of the world's population, affecting approximately one billion people of different ethnicities and ages. This widespread prevalence of hypovitaminosis D occurs due to insufficient exposure to solar radiation and vital environmental factors crucial to vitamin D synthesis in the skin [17].

A reliable indicator of vitamin D status is regarded as the 25-hydroxyvitamin D concentration. The United States Endocrine Society guidelines define vitamin D deficiency as a 25(OH)D concentration of less than 50 nmol/L, while 50–75 nmol/L is considered insufficient. The etiology of vitamin D deficiency can be attributed to a multitude of factors, including aging, inadequate engagement in outdoor activities, excessive use of sunscreen, inadequate vitamin D supplementation, obesity, high latitudes, and diet [14].

A four-year follow-up cohort study was conducted as part of the Irish Longitudinal Study of Ageing (TILDA) (aged ≥ 50 years) to ascertain the relationship between vitamin D, depression and other variables. The study incorporated a range of demographic and health-related factors, including age, sex, body mass index, smoking, alcohol use, physical activity, chronic disease burden and cardiovascular disease. The findings indicated that individuals with vitamin D deficiency exhibited a heightened likelihood of experiencing depression, at a rate 75% higher than that observed in individuals with adequate vitamin D levels [19]. Furthermore, an inverse relationship between plasma 25(OH)D3 and depression symptoms and major depressive disorder (MDD) was identified in cross-sectional and cohort studies [20–22].

### Vitamin D in the Serotonin Pathway: Gene Expression Modulation

**Fig. 1:**
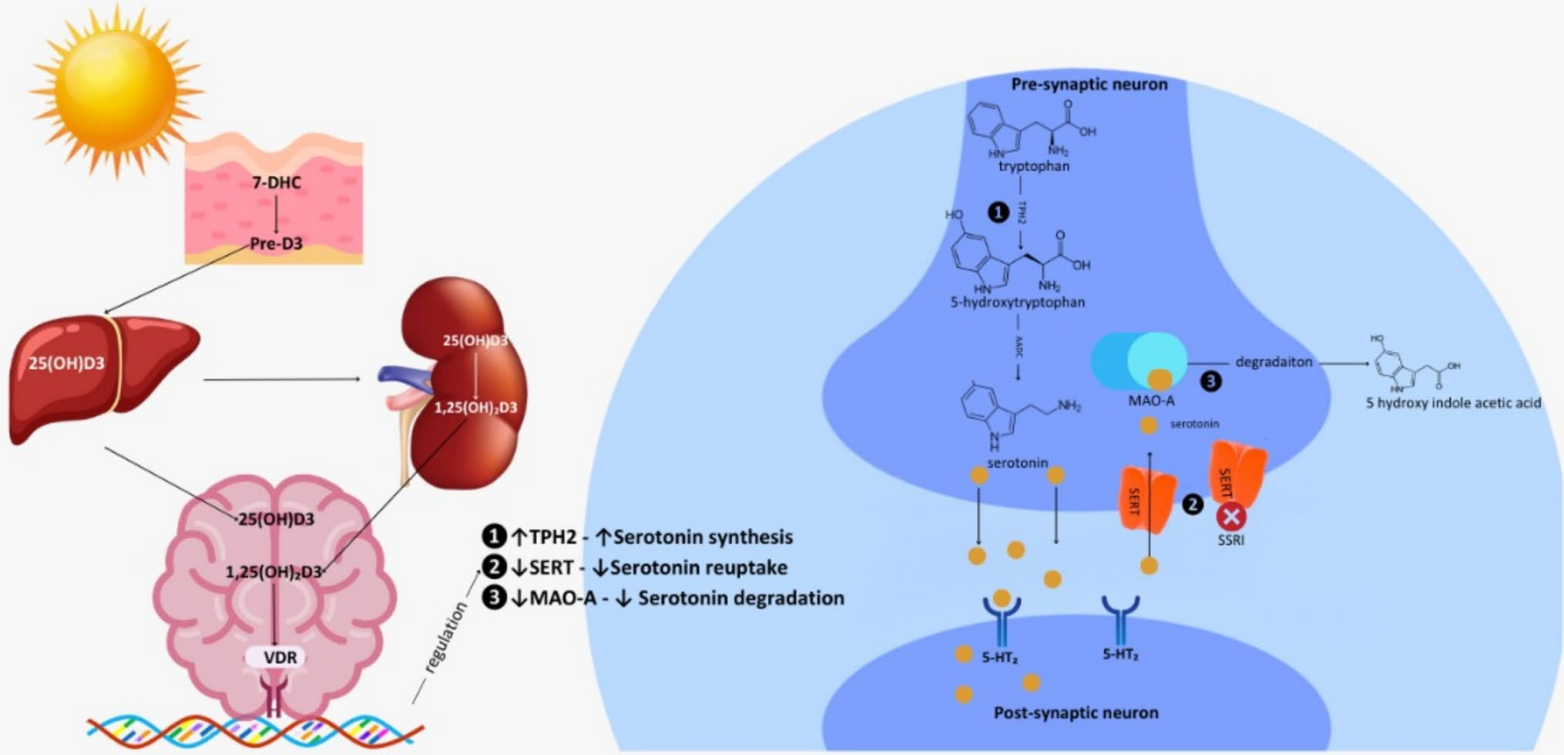
Vitamin D in the serotonin pathway. 1,25(OH)2D3:dihydrocyclolecalciferol; 5HT2:5-hidroksitriptamin receptor; 7-DHC:7-Dehydrocholesterol; 25(OH)D3:25-hydroxyvitamin D; MAO-A:Monoamine oxidase A; SERT: serotonin transporter inhibitor; TPH2: Tryptophan hydroxylase 2; VDR: Vitamin D receptor

The neurotransmitter serotonin is produced from tryptophan and released throughout the nervous system, specifically the brain [2, 23]. Three crucial enzymes/transporter proteins that regulate serotonin levels and dynamics within the central nervous system (CNS) are tryptophan hydroxylase type 2 (TPH2), the serotonin transporter (SERT), and monoamine oxidase-A (MAO-A) [24, 25]. Its synthesis involves two main steps: the initial rate-limiting step is the hydroxylation of tryptophan to 5-hydroxytryptophan (5-HTP) by tryptophan hydroxylase (TPH), which occurs in both the CNS and peripherally. Following this step, the 5-HTP undergoes decarboxylation to form serotonin, also known as 5-hydroxytryptamine (5-HT), which is catalyzed by aromatic amino acid decarboxylase [2, 23]. The interrelationship between vitamin D and serotonin is illustrated in Fig. 1.

The enzyme TPH, which is accountable for the limiting step in the production of serotonin by vitamin D, is markedly induced by the metabolically active vitamin D metabolite 1,25(OH)2D in serotonergic neuron [10, 11].

Vitamin D also has a significant function in regulating central and blood serotonin concentration by controlling the transcription of TPH 1 and TPH 2 genes [26]. It is possible that serotonin concentrations are regulated, at least in part, by 1,25(OH)2D, which is the active endocrine metabolic product of vitamin D. Jiang et al. conducted in vivo experiments that showed a significant increase in TPH2 mRNA in the prefrontal cortex of rats treated with chronic 1,25(OH)2D administration [2].

An accumulation of clinical and experimental evidence indicates that serotonergic neurotransmission dysregulation may be a contributing factor in the aetiology of a range of psychiatric disorders, including attention deficit hyperactivity disorder, autism, and depression. Additionally, there is a growing recognition of the potential involvement of serotonergic pathways in the development of antisocial, obsessive–compulsive, and psychotic behaviors, as well as suicidal ideation and attempts [27]. The effects of serotonin on the brain have been observed to influence a number of cognitive processes, including executive function, sensory gating, and levels of prosocial behavior. Crucially, disorders of these actions often display serotonin deficiency in their pathological manifestation [2]. Adequate serotonin production is necessary for proper serotonergic function, with TPH activity predominantly controlling this process [27].

## Vitamin D and Serotonin in The Treatment of Psychiatric Disorders

Vitamin D is crucial for maintaining optimal brain and nervous system health, as it serves as an essential neurosteroid hormone involved in brain development, neuroplasticity, and neuroimmunomodulation. Consequently, it may impact mood regulation indirectly [16].

Vitamin D receptors are present in a number of different tissues, including the prefrontal cortex, cingulate cortex, thalamus, hypothalamus, hippocampus and substantia nigra. It is postulated that these areas are implicated in the underlying neurobiology of depression. The widespread distribution of the vitamin D-activating enzyme 1-α-hydroxylase across numerous brain regions, including amygdala neurons and hypothalamic glial cells, provides further evidence of the interrelationship among vitamin D and neuropsychiatric illnesses. The active form of vitamin D is capable of crossing the blood–brain barrier, suggesting that vitamin D may have a direct or indirect role in brain and cognitive functions, as reported by Akpinar and Karadag (2022) [28].

Vitamin D insufficiency may modify the accessibility of certain cholinergic, dopaminergic, and noradrenergic neurotransmitters implicated in depression [8]. The correlation between anomalous serotonin levels during the process of development and in adults was investigated, as well as the association found in a diverse range of behavioural disorders, including Attention-deficit/hyperactivity disorder (ADHD), autism, bipolar disorder, depression and schizophrenia, alongside antisocial, obsessive–compulsive and suicidal behaviors. It was observed that vitamin D deficiency and low central serotonin concentrations were common denominators in a multitude of neuropsychiatric disorders [2]. The clinical symptoms of each disorder may differ, yet all display physical, cognitive, and behavioral symptoms that cause considerable disruptions to daily activities and are arduous to manage [16].

Several hypotheses exist that explain the neurological effects of calcitriol without exclusively relying on activating gene expression of the tyrosine hydroxylase enzyme. Calcitriol has been observed to increase choline acetyltransferase activity while decreasing acetylcholinesterase activity, thus enhancing cholinergic function. Additionally, it serves as a potent enhancer of nerve growth factor (NGF) and glial-derived neurotrophic factor (GDNF) while increasing neurotrophin 3 (NT-3) activity and decreasing neurotrophin 4 (NT-4) activity. Recent studies indicate that NGF, NT-3, and GDNF may be involved in the development of depression and schizophrenia. Moreover, it contributes to the brain's protection against oxidative degeneration by elevating glutathione levels, a crucial antioxidant, by amplifying c-glutamyl transpeptidase gene expression [29]^.^

A meta-analysis of 41 randomized controlled trials (RCTs) has demonstrated that vitamin D supplementation has a moderate to marginal influence on depressive symptoms. Additionally, the meta-analysis discovered that the impact was more pronounced in studies where vitamin D was administered above 2000 IU (50 μg). Despite inconsistent findings, maintaining an adequate calcidiol concentration is typically viewed as advantageous for maintaining good health [8].

### Major Depressive Disorder (MDD)

MDD is a common psychiatric diagnosis that typically manifests during adolescence. The condition is typified by a constellation of psychopathology, encompassing depressive affect, reduced interest or satisfaction in daily activities, feelings of guilt or self-reproach, suicidal ideation, psychomotor retardation, impaired cognitive function, sleep disturbances, and altered appetite. These symptoms affect an estimated many people globally [18, 30]. Depressive episodes are known to result in suffering for individuals, decreased productivity, heightened health costs, and increased risk of suicide [31]. The causal mechanisms and pathogenesis of MDD, however, have only been partially understood historically. Approximately 35% of MDD can be attributed to its heritability [30, 32]. Depression is most common in individuals over the age of 55 and affects females by almost 50% more than males. The vast number of individuals affected by this condition, and the approximately 700,000 annual suicide-related deaths, highlight the significant impact it has on public health [33].

As delineated in the Diagnostic and Statistical Manual of Mental Disorders (DSM-5), a diagnostic classification of major depression necessitates the manifestation of a minimum of five symptoms over a span of at least two weeks. At least one symptom of MDD must include depressed mood or anhedonia. Other potential symptoms may include secondary changes in appetite or body weight, sleep disturbances, psychomotor agitation, fatigue, diminished cognitive abilities, feelings of worthlessness, and excessive guilt [28]. The etiology of MDD involves immune and environmental changes, as well as complex behavioral and neurochemical factors and environmental stress [32].

Antidepressants, psychotherapies, and their combinations are frequently administered in everyday medical care. However, these treatments only produce modest results, and over 50% of patients do not achieve the desired effect size [34, 35].

The contemporary lifestyle and dietary habits have been identified as contributing factors to a significant rise in cases of depression. This has prompted the question as to whether there is a correlation between diet and the prevalence of this condition [33]. The influence of dietary and social habits on mental well-being is a vast and complex subject, encompassing areas such as the brain-gut axis and nutrigenomics. The extant literature provides compelling evidence that an individual's dietary habits, exercise regimen, and sleep patterns play a pivotal role in the efficacy of a given treatment [33, 34].

The potential mechanism linking low levels of vitamin D and MDD is rooted in the possibility that vitamin D can disturb the production and discharge of crucial neurotransmitters like dopamine and serotonin, thereby disrupting mood regulation [18, 34]. Vitamin D exerts its influence on the brain through its binding to vitamin D receptors, which are extensively dispersed in the prefrontal cortex, cingulate cortex, and limbic system. Vitamin D, acting as a neurosteroid hormone, may have a regulatory function in neurotransmission, neuroprotection, and neuroimmunomodulation [18]. Additionally, vitamin D metabolites may play a part in the development of MDD by influencing inflammatory pathways, given the strong link between inflammation and MDD [18, 34].

In the study conducted by Khoraminya et al. (2013) [36] 42 patients diagnosed with MDD were divided into two groups: one received 20 mg fluoxetine for eight weeks and the other received 1500 IU of vitamin D in conjunction with 20 mg fluoxetine. The two-way repeated-measures analysis of variance showed that depression severity decreased significantly after intervention with a significant difference between the two groups. Analysis of covariance for depression severity adjusted for baseline values at weeks 2, 4, 6 and 8 showed that the vitamin D–fluoxetine combination was significantly better than fluoxetine alone from the fourth week of treatment. However, the administration of fluoxetine as a standalone agent was also found to engender a therapeutic effect, with a significant reduction in depression severity becoming apparent from the second week of treatment onwards.

Vellekkatt et al.'s double-blind, RCT included 46 MDD patients randomized to standard treatment or a parenteral dose of 3,00,000 IU vitamin D added to standard treatment. At the conclusion of the 12-week intervention, Vellekkatt et al. (2020) [37] reported that the provision of 300,000 IU of vitamin D was efficacious in enhancing depression symptom rating scores, while also significantly ameliorating both the quality of life and clinical disease severity.

In a double-blind RCT, 59 participants with serum 25(OH)D levels below 20 ng/ml were randomly assigned to one of two groups: a treatment group that received 60,000 IU of vitamin D supplementation every five days for 12 weeks, and a control group that received a placebo. No significant correlation was identified between 25(OH)D levels and depression scores throughout the study period. It is noteworthy that the escitalopram doses administered to the placebo group were 4 mg/day higher than those provided to the vitamin D treatment group [38].

Postolache et al. (2020) conducted a study comparing the mRNA expression of the VDR gene in the anterior cingulate cortex and dorsolateral prefrontal cortex between individuals who died by suicide due to depression and controls who died of non-psychiatric causes. They found higher VDR expression among the suicide group compared to controls, and this up-regulation of VDR mRNA levels is partly attributed to vitamin D deficiency [39].

### Anxiety

According to the World Health Organization, as of 2017, 3.6% of the worldwide population (approximately 264 million) suffers from anxiety disorders. Moreover, women (4.6%) are more affected by anxiety than men (2.6%) [16]. It has been reported that the prevalence of depressive and anxiety disorders and the associated disease burden increased significantly (more than 25% in the first year of the pandemic) worldwide during the COVID-19 pandemic [30].

Vitamin D is neuroactive and important for brain development. The ligand has been observed to bind to receptors that are commonly expressed in both neuronal and glial cells. Vitamin D is involved in brain processes like immunomodulation, neuroinflammation and neurotrophic signalling [16].

Several hypotheses have been proposed regarding anxiety disorders. One hypothesis posits that the anterior cingulate cortex (ACC) serves as the nexus for bidirectional connections that modulate both fear and anxiety. These pathways are thought to be influenced by the hippocampus, amygdala and ventromedial prefrontal hypothalamus. It is further suggested that a mismatch between these areas can lead to anxiety disorders. Another assumption is that a malfunction of the hypothalamic–pituitary–adrenal (HPA) axis, which also contributes to the pathophysiology of depression, leads to upregulation of vitamin D receptor expression in these areas and increased vitamin D activity due to the presence of the enzyme 1-alpha-hydroxylase. Abnormalities in the HPA axis have been reported in lack of vitamin D. This evidence is supportive of the association with vitamin D and anxiety [16, 28]. In an RCT investigating the effect of vitamin D supplementation on anxiety, 6 months of 1600 mg vitamin D supplementation resulted in improvement in anxiety symptoms [28]. A systematic review of 20 RCTs indicated that supplementation with vitamin B and/or vitamin D might be a well-tolerated and effective adjunctive approach for alleviating symptoms of anxiety and depression, depending on patients' nutritional biomarkers and clinical status [40]. Zhu et al. [6]. investigated the effects of vitamin D deficiency (25(OH)D < 75 nmol) in a RCT involving 158 people with MDD. The intervention group received a daily supplementation of 1,600 mg of vitamin D for 6 months, while the control group remained unchanged. Although no significant change in depressive symptoms was observed, the treatment group showed a significant improvement in anxiety symptoms compared with the other group.

### Bipolar Disorder

The first reports of what is now known as bipolar disorder (BD) date back to ancient and middle-age writers, and others to the eighteenth century. In the 19th, the Parisian scientist Jules Baillarger (1809–1890), based on his clinical trials at the Hôpital Saltpêtrière, first presented the concept of bipolar mania, consisting of phases of agitation and depression in presentation given at the Académie de Médecine in 1854 [41, 42]. BD is currently defined as a severe psychiatric condition characterized by episodes of depression, mania, or a combination thereof. The disorder occurs in around 1–2% of the public. While bipolar I disorder occurs both sexes evenly, bipolar II disorder is more frequent in the female gender. The different subtypes of bipolar disorder are classified according to the severity and frequency of the episodes. Besides bipolar I and II, other subtypes include fast cycling (more than four manic, depressive, hypomanic, or mixed episodes in one year) and cyclothymia (hypomanic and subdepressive symptoms over two years) [43].

Unhealthy lifestyles and comorbid medical conditions are typical in people with BD. Dietary factors may play a role in the development of depression and anxiety, according to a growing body of research. Consequently, nutritional interventions are regarded as a promising approach in the treatment of BD. A recent meta-analysis indicates that dietary intake or supplementation with n-6 and n-3 fatty acids, particularly n-3, is related to enhanced BD symptoms. Additionally, seafood, vitamin D, folic acid, and zinc appear to be beneficial for BD patients [44]. Some epidemiological researchs indicate that ω−3 insufficiency might be a risk factor for BD: plasma DHA has been reported to be reduced significantly in individuals with BD [45].

Individuals diagnosed with BD have an approximate mortality rate that is approximately twice that of the general population. A significant proportion of these deaths are attributed to suicide, resulting in a lifetime risk of approximately [46]. Suicidal thoughts are commonly seen in people with BD and have been associated with both low levels of omega-3 s and low levels of serotonin in the brain [47, 48]. RCTs have shown that supplementation with even a few grams of eicosopentanoic acid (EPA) and docosapentaenoic acid (DHA) improves depression, suicidal ideations and behaviours [49, 50]. In addition, 58% of those who attempted suicide were found to be vitamin D deficient; these individuals had significantly lower vitamin D levels compared to healthy individuals and patients with depression but no suicidal tendencies [51]. In parallel, shorter daily sunshine hours have also been associated with higher suicide rates regardless of season [52, 53].

### Schizophrenia

Schizophrenia is a complex and poorly comprehended brain disorder, typified by abnormalities in high-level functions associated with perception, cognitive-communication, strategic planning, and motivation. The illness is typified by the emergence of hallucinations, delusions, impaired cognitive processes, and a constellation of adverse symptoms, including diminished emotional expression and communication difficulties [54]. Schizophrenia is known to affect approximately 24 million people worldwide and is also recognised as a 'neurodevelopmental' disorder, in which genetic or environmental influences are predicted to occur during early brain development, in utero or shortly after birth [55, 56].

Deficiency of vitamin D is a common occurrence in individuals diagnosed with ASD, ADHD, BD, schizophrenia, and impulsiveness [51, 57]. Consequently, individuals at risk of, or already diagnosed with, one of these disorders may well benefit from vitamin D supplementation [58]. Latest researchs have shown that neonatal vitamin D deficiency increases the risk of schizophrenia in later life [59]. McGrath et al. (2004) reported that vitamin D supplementation in the first year of life was associated with a 77% decrease in the incidence of schizophrenia. It has been reported that vitamin D deficiency and insufficiency are common in schizophrenia patients, and preclinical studies have shown that this deficiency may lead to schizophrenia by affecting dopamine-related genes. It has also been highlighted that vitamin D deficiency in adults may affect cognitive function by disrupting neurotransmitter balance and is associated with obesity [59].

### Obsessive–Compulsive Disorder (OCD)

The presence of obsessions and/or compulsions defines obsessive–compulsive disorder (OCD). Obsession is a recurring thought or image that is experienced as undesirable and is usually associated with anxious feelings. Compulsions are recurring behaviours or mental actions that individuals perform in order to meet strict rules or to achieve a sense of completion in response to an obsession [60]. OCD usually begins early in life and is long-lasting. In the National Comorbidity Survey Replication (NCS-R) study, almost a quarter of men had an onset before the age of 10 [61]. In women, onset is usually in adolescence, but some women may have OCD in the antenatal or postnatal period [62].

Cognitive behaviour therapy and/or medication with SSRIs, the tricyclic antidepressants, are recommended for OCD [63–65]. The limited efficacy and potential adverse reactions of current treatments have led to a look for alternative strategies. Several nutritional deficiencies are known to be present in individuals with mental disorders. Thus, supplements are considered to be an effective treatment [66].

There are a number of potential relationships between vitamin D and the pathophysiology of OCD. One is through the relationship between calcitriol and ratio of tyrosine to tryptophan hydroxylase. Tyrosine hydroxylase is the critical enzyme in the synthesis of epinephrine, norepinephrine and dopamine, and tryptophan hydroxylase is the essential enzyme in the synthesis of serotonin. 1,25(OH)2D regulates the levels of these two enzymes [27, 67]. It is therefore plausible to suggest that deficiencies in vitamin D may play a contributory role in the aetiology of OCD by interfering with the pathways of serotonin and catecholamine synthesis. Another link between vitamin D and OCD is related to the protective effect of vitamin D on the nervous system. Several researchs have demonstrated the role of free radicals and significantly elevated levels of nitric oxide in the development of OCD [68]. Vitamin D has antioxidant properties that inhibit an enzyme (inducible nitric oxide synthase) that is necessary for nitric oxide synthesis. For this reason, vitamin D deficiency may play a role in the aetiology of OCD by impairing neuroprotection [66, 69].

### Attention Deficit Hyperactivity Disorder (ADHD)

ADHD is a neurological condition characterised by symptoms of inattentiveness, overactivity and impulsivity [70]. Impairments in executive functioning, the skills of planning, organising, paying attention, managing time, shifting focus, remembering details or doing things based on previous experience, and multitasking are also characteristic of ADHD [71].

Although there is no completely curative treatment for ADHD, some evidence-based interventions can significantly reduce its symptoms and associated disorders. Medications are both effective and well tolerated in the management of ADHD. Neurocognitive interventions, behavioral therapy, dietary restriction approaches, and nutritional supplements are also used [72]. Western-style diets high in artificial colours, preservatives, fat and refined sugars have been associated with an at risk of ADHD [73]. In opposition, the Mediterranean diet [74]. or some other patterns of diet rich in fibre, folate and ω−3 fats have been reported to be adversely associated with ADHD [75].

The results of a number of observational trials indicate the significance of vitamin D in the aetiology of childhood mental defects. A reduction in mean 25(OH)2D levels has been demonstrated in children and adolescents with ADHD compared to controls [76–78]. In a recent study, an 8-week vitamin D3 intervention (50,000 IU per week) in children with ADHD not only increased serum levels of this vitamin, but also reported that its use during feedback may elicit more favourable clinical and electrophysiological outcomes [79].

## Use of SSRIs in Psychiatric Disorders

In the pathology of various mood disorders, serotonin is widely implicated [80]. Serotonin neurons make up a tiny proportion of brain neurons, but they signal almost all brain regions. SSRIs inhibit serotonin reuptake to increase stimulation of postsynaptic serotonin receptors. The serotonin system is the target of many therapeutic drugs, including SSRIs, which have been approved by the US Food and Drug Administration for the treatment of MDD since 1987 and have indications for a variety of mental health conditions, including anxiety, compulsions and eating disorders [81–83].

Some studies have indicated a significant correlation between vitamin D treatment and clinical improvement in patients undergoing standard pharmacological therapy (SSRIs). This evidence provides support for the hypothesis that vitamin D may serve as an effective adjunctive treatment [36, 84, 85].

The use of SSRIs can lead to reduced bone density [86]. There is an elevated prevalence of osteoporosis, particularly among patients undergoing prolonged SSRI therapy [87]. The precise mechanism by which SSRI-induced bone loss occurs remains unclear. However, there is some data to suggest that these drugs may have a detrimental impact on bone matrix deposition [88, 89]. Furthermore, there is evidence to suggest that chronic SSRI use may be associated with the development of osteoporosis, with the inhibition of bone matrix deposition being a potential mechanism [88, 90]. In the study examining the association between SSRI use and bone mass density (BMD), malnutrition, which can occur in depressed people prescribed SSRIs, was also found to be associated with reduced BMD. More than half of SSRI users and non-users were reported to be deficient in calcium (p = 0.07), vitamin D (p < 0.01) and potassium (p = 0.05).This difference was statistically significant only for vitamin D [91].

A substantial body of in vivo and clinical evidence on the effects of SSRIs on bone health indicates that the mechanism of action is complex and that the interpretation of these findings is challenging [87, 90]. Although a considerable number of the consistent conclusions indicate that SSRI utilization may be deleterious to bone and cause bone density to decrease, a causal relationship cannot be definitively established. Evidence that depression itself causes bone loss, both because of inherent biological changes and because of unhealthy diet and lack of exercise ect. that often accompany depression, supports depression as an illness. In addition, further studies are needed to clearly establish the relationship between SSRI use and bone and to confirm recent promising animal reports of potential prevention/treatment of bone loss [86].

## The Potential of Vitamin D to Reduce the Need for SSRIs: A Comparison and Evaluation

The exploration of alternative or complementary therapies has gained significant attention due to the limitations and side effects associated with conventional pharmacological treatments such as SSRIs. In contrast to SSRIs, vitamin D has been shown to have a milder and more tolerable adverse effect profile in the treatment of depression [92]. This is due to a number of factors, including its lower propensity to cause side effects, the potential for reduced adverse interactions with other medications, and a lower prevalence of comorbidity in older adults. Consequently, Vitamin D was proposed as a potentially safer adjuvant treatment option for the management of depressive symptoms.Moreover, while long-term use of SSRIs may result in adverse effects such as decreased bone density [86], Vitamin D has been shown to promote bone health [15]. Furthermore, the occurrence of adverse effects such as gastrointestinal disorders and sexual dysfunction, which have been frequently reported in SSRIs users, has not been consistently observed in Vitamin D supplementation [93–95].

In terms of financial implications, a body of research has indicated that Vitamin D supplements tend to be more cost-effective in comparison to pharmaceutical interventions, whilst also offering accessibility to a broader demographic of users. This cost-effectiveness is particularly salient for chronic conditions, such as depression, that necessitate prolonged treatment [92, 96].

In relation to the duration of the therapeutic effect, it has been established that the effects of SSRIs generally manifest within a period of 4–6 weeks. This phenomenon can be attributed to alterations in neurotransmitter equilibrium, which arise from the inhibition of serotonin transporters. Conversely, Khoraminya et al. (2013) demonstrated that the combination of fluoxetine and Vitamin D yielded substantial outcomes within a mere four weeks, thereby providing clinical evidence that the regulatory effects of Vitamin D on serotonin synthesis can be discerned more expeditiously [36].

It is hypothesised that vitamin D should be considered as an agent that enhances the effect of SSRIs rather than leading to their complete elimination. As demonstrated in the literature, vitamin D supports the goal of SSRIs to increase serotonin concentration, and this combination offers faster and stronger therapeutic effects [40, 84, 85]. Furthermore, it is stated that this combination allows the dose of SSRIs to be reduced and side effects to be minimised. Consequently, the evaluation of vitamin D as a supportive agent in the treatment of depression may contribute to the development of safer and more effective treatment strategies.

## Conclusions

In 2019, it was estimated that 970 million people worldwide were living with a mental disorder, with anxiety and depressive disorders being the most prevalent [56]. However, the global pandemic caused by the SARS-CoV-2 virus resulted in a significant increase in the number of individuals affected by these illnesses in 2020. Preliminary estimates suggest a 26% and 28% rise in anxiety disorders and MDD, respectively, over the course of a year [97]. Concomitant with the global prevalence of mental disorders, approximately 1 billion people worldwide are vitamin D deficient. Vitamin D has been shown to have immunomodulatory, neuroprotective and neurotrophic properties, although the majority of studies have demonstrated a favourable impact, with outcomes remaining inconclusive. The causes of conflicting results in trials examining vitamin D status have been suggested to be differences in cut-off for vitamin D sufficiency, differences in the groups in which the trials were conducted, and differences in the measurement tools used.In addition to the fact that vitamin D deficiency causes neuropsychiatric disorders or that dietary intake/supplementation has a role in the treatment of neuropsychiatric disorders alone, it has been shown to have an adjuvant role in patients treated with SSRIs.

This review highlights the complex interplay between vitamin D and serotonin synthesis and the potential to enhance the efficacy of SSRIs in the treatment of various neuropsychiatric disorders.The synergistic mechanism is likely based on vitamin D altering the levels of TPH2, SERT and MAO-A, which play critical roles in regulating serotonin level in the brain.

The augmentation of serotonin synthesis by vitamin D may act in a complementary manner to SSRIs, whose mechanism of action is predominantly the inhibition of serotonin reuptake.This dual mechanism is hypothesised to result in the optimisation of serotonergic activity and the enhancement of clinical outcomes.In comparison to SSRIs, vitamin D offers several advantages. These include its immunomodulatory, neuroprotective and anti-inflammatory properties, which extend beyond the regulation of serotonin and address other pathways implicated in the pathophysiology of mental health disorders.Moreover, the tolerability of vitamin D supplementation is generally high, with a favourable side-effect profile in comparison to SSRIs, which are associated with adverse effects such as sexual dysfunction, weight gain, and an increased risk of osteoporosis with long-term use. However, the literature contains inconsistent findings, indicating a necessity for further research into optimal dosing, population-specific effects, and the duration of supplementation required to achieve sustained benefits.The role of vitamin D may vary across different psychiatric conditions, reflecting the heterogeneity of serotonergic dysfunction in disorders such as anxiety, depression, and OCD.

For instance, while serotonin dysregulation is a central feature of all three disorders, the mechanisms by which vitamin D influences each disorder may differ due to different neurobiological and environmental interactions. Investigation of these disorder-specific pathways has the potential to provide targeted therapeutic strategies. It is recommended that future studies investigate the potential side effects of vitamin D, particularly those associated with high-dose or long-term supplementation, such as hypercalcaemia or drug interactions. In addition, a more comprehensive investigation of the wider effects of vitamin D deficiency on mental health, particularly in vulnerable populations, may further elucidate its role as a preventive and therapeutic agent in psychiatry. In conclusion, the evidence presented in this review suggests that vitamin D has the potential to be a valuable adjunct to traditional pharmacotherapy for neuropsychiatric disorders. However, it is crucial to recognise that addressing the existing gaps in the current evidence through well-designed longitudinal studies is paramount to the full integration of vitamin D into clinical practice as part of a comprehensive, personalised approach to mental health care.

## Data Availability

No datasets were generated or analysed during the current study.
